# Coronary Microvascular Dysfunction and Vasospastic Angina—Pathophysiology, Diagnosis and Management Strategies

**DOI:** 10.3390/jcm14041128

**Published:** 2025-02-10

**Authors:** Joanna Abramik, Mark Mariathas, Ioannis Felekos

**Affiliations:** 1 Bristol Heart Institute, University Hospitals Bristol and Weston NHS Foundation Trust, Terrell Street, Bristol BS2 8ED, UK; joanna.abramik@uhbw.nhs.uk (J.A.); mark.mariathas@uhbw.nhs.uk (M.M.); 2 Department for Health, University of Bath, Claverton Down, Bath BA2 7AY, UK

**Keywords:** coronary microvascular dysfunction, vasospastic angina, invasive coronary function testing, personalised medical therapy, coronary sinus reducer

## Abstract

Coronary artery disease is one of the leading public health problems in the world in terms of mortality and economic burden from the disease. Traditionally, the focus of research and clinical pathways leading to the diagnosis and treatment of coronary artery disease was on the more common variant of the disease resulting from atherosclerosis in the epicardial coronary arteries. However, coronary microvasculature, representing the vast majority of the total heart circulation, has the greatest influence on overall coronary resistance and, therefore, blood flow. Coronary microvascular dysfunction (CMD), characterized by structural or functional abnormalities in the microvasculature, significantly impacts myocardial perfusion. Endothelial dysfunction results in inadequate coronary dilation during exercise or spontaneous spasm in the microvasculature or epicardial arteries. A significant proportion of people presenting for coronary angiography in the context of angina have unobstructed epicardial coronary arteries yet are falsely reassured about the benign nature of their condition. Meanwhile, increasing evidence indicates that patients diagnosed with CMD as well as vasospastic angina (VSA) face an increased risk of Major Adverse Cardiovascular Events (MACEs), including death. The aim of this review is to outline the current practice with regard to invasive and non-invasive methods of CMD and VSA diagnosis and assess the evidence supporting the existing treatment strategies. These include endotype-specific pharmacological therapies, a holistic approach to lifestyle modifications and risk factor management and novel non-pharmacological therapies. Furthermore, the review highlights critical gaps in research and suggests potential areas for future investigation, to improve understanding and management of these conditions.

## 1. Introduction

Ischaemic heart disease is a leading cause of global mortality and morbidity. Research has traditionally focused on epicardial coronary artery stenosis due to atherosclerosis. However, large studies show that only 35–60% of patients with angina symptoms have obstructive coronary artery disease detected via elective angiography [1,2]. The European Society of Cardiology’s (ESC) updated definition of Chronic Coronary Syndromes (CCSs) emphasizes recognizing the impact that structural and functional alterations to the microvasculature may have on transient episodes of myocardial hypoperfusion, which may manifest as angina or angina-equivalent symptoms [3].

Patients with angina and unobstructed coronary arteries (ANOCAs) have previously been reassured about their condition’s benign nature [4], but evidence now indicates an unfavourable prognosis, including a higher risk of Major Adverse Cardiovascular Events (MACEs) and mortality [2,5,6,7,8]. The condition not only has a great impact on health resource utilisation, with increased costs relating to repeat hospitalisations, invasive catheterisation and anti-ischaemia drug treatment [9], but also has a large functional and macroeconomic impact relating to reduced quality of life of the affected patients [10,11].

Despite international guidelines recommending invasive coronary functional testing for persistently symptomatic ANOCA patients [3,12], barriers to adoption remain, such as the need for procedural expertise, potential complications, additional time required to undertake the procedure and its costs. However, there is compelling evidence as to the cost-effectiveness of this testing [13], and the benefits that stratified medical therapy according to the endotype of the disease has on patients’ symptom burden and quality of life [14]. The purpose of this paper is to summarise the existing evidence with regard to the diagnosis and management of coronary microvascular dysfunction and vasospastic angina as important ANOCA endotypes.

## 2. Normal Coronary Vascular Structure and Function

The coronary vascular tree consists of two compartments: the epicardial, which includes arteries and veins, and the microvascular, comprising arterioles, capillaries and venules. Epicardial coronary arteries are conduit vessels that contribute less than 5% resistance to blood flow. Coronary arterioles, which account for about 60% of resting vascular resistance, are crucial for regulating coronary blood flow. These arterioles branch into smaller ones that penetrate the myocardium and lead to capillaries. While capillaries represent only 25% of resting vascular resistance (with venules and veins making up the remaining 10%), they contain 90% of myocardial blood volume and are key sites for metabolic exchange with myocardial tissue [15].

Large arterioles and coronary arteries are primarily controlled by the endothelium. Shear stress from blood flow triggers nitric oxide (NO) release, causing vascular smooth muscle cells (VSMCs) to relax in a process called flow-mediated vasodilation [15]. Medium-sized arterioles respond to changes in blood pressure through Ca^2+^-mediated VSMC vasoconstriction, an endothelium-independent myogenic response [16]. Small arterioles are influenced by local metabolites, like adenosine and carbon dioxide, which diffuse into VSMCs and cause vasodilation and increased perfusion [15]. Additionally, the autonomic nervous system affects coronary blood flow by altering metabolite release and directly stimulating alpha and beta-adrenoceptors on VSMCs. Together, these mechanisms regulate vasomotor tone to align myocardial perfusion with demand [17].

## 3. Classification and Pathophysiology of ANOCA

The term ANOCA is used to describe patients who present with angina in the context of non-obstructive coronary arteries. Of these, depending on the type of the test performed, only 10–30% of patients have demonstrable ischaemia on stress testing (termed Ischaemia in Non-Obstructed Coronary Arteries (INOCA)) [6,18,19]. ANOCA/INOCA can be further subdivided into endotypes: (i) coronary microvascular dysfunction (CMD), which can be further subdivided into structural and functional CMD (either endothelium-dependent or independent); (ii) vasospastic angina (VSA), a result of epicardial coronary spasm or microvascular spasm; (iii) both CMD and VSA; (vi) non-cardiac chest pain [20]. A recent meta-analysis has shown that, amongst patients presenting with cardiac chest pain and unobstructed coronary arteries, the prevalence of CMD was 41% (with women more likely to be affected), and the overall prevalence of epicardial or microvascular spasm was 49%, with both sexes equally affected. Interestingly, in three studies within the meta-analysis, which evaluated a total of 541 patients for both CMD and spasm, the prevalence of CMD alone was 23%, coronary spasm alone (either epicardial or microvascular) 19%, and coexistent CMD and coronary vasospasm 23%, which further supports the need for complete functional assessment of coronary physiology in patients presenting with ANOCA [21].

### 3.1. Structural CMD

Structural CMD comprises a disorder of microvascular circulation defined as reduced coronary flow reserve (CFR) in association with an increased minimal microvascular resistance (MMR) [7]. The proposed pathophysiology includes changes to vessel architecture, such as capillary rarefaction or arteriolar obliteration [7]. However, patients with structural CMD have also been shown to exhibit endothelial dysfunction, which leads to diminished peak coronary blood flow augmentation and increased demand during exercise [22]. Patients with structural CMD often have established coronary disease risk factors, such as diabetes or hypertension, and show an augmented blood pressure response to exercise [18], which further increases myocardial oxygen demand and predisposes them to ischaemia. It has been proposed that those patients benefit from lifestyle modification (e.g., weight loss or smoking cessation) and treatment leading to afterload reduction and vascular remodelling, for example, with angiotensin-converting enzyme inhibitors (ACEis) or statins [7,22].

### 3.2. Functional CMD

Historically, the pathophysiology of CMD was considered to result from high vascular resistance during increased demand for vasodilation (which nowadays is termed structural CMD). Interestingly though, these patients represent less than 50% of those with impaired CFR [22]. Functional CMD appears to result from increased demand for myocardial oxygenation at rest or disordered coronary autoregulation. This leads to sub-maximal vasodilation in resting conditions, with the inability of those patients to further augment coronary blood flow in periods of stress, leading to an overall reduced CFR in the presence of normal or decreased MMR [7]. This still has important clinical significance due to the impact of reduced CFR on prognosis [7,8]. It has been hypothesised that functional CMD can act as a precursor to structural CMD, as chronically raised coronary blood flow may precipitate structural vascular changes, a process common in other vascular beds, such as renal or pulmonary [18]. This association has not been proven and no disease-modifying therapies have been developed specifically for functional CMD, although, in theory, therapies modulating cardiac metabolism could be of benefit [7].

### 3.3. Vasospastic Angina (VSA)

In contrast to CMD, which results from impaired vasodilation of the microvasculature, vasospastic angina is a disorder resulting from vasoconstriction of the coronary arterial system. VSA typically involves the epicardial coronary arteries, but microvascular spasm, resulting from vasoconstriction of pre-arterioles and arterioles, has also been described [23], and both entities can co-exist. VSA typically occurs at rest and in a circadian pattern but may be triggered by catecholamine surges after exercise or stress [24]. VSA occurs spontaneously, but it can also be induced through acetylcholine (ACh) provocation testing in the cardiac catheterisation laboratory. Epicardial VSA has been attributed to abnormal reactivity of the epicardial arteries to vasoconstrictive stimuli affecting the VSMCs [23]. This is especially profound in the context of endothelial dysfunction—in a normally functioning endothelium, vasoactive agents, like ACh or histamine, lead to vasodilation through NO release, whereas, in dysfunctional endothelium, these agents lead to vasoconstriction through activation of VSMCs [25]. In microvascular spasms, there is an increased local release of vasoconstrictive substances, associated with an increased susceptibility of VSMCs, or an abnormal activity of sympathetic tone [26]. Cigarette smoking is the primary risk factor for VSA, with hypertension, diabetes and hypercholesterolemia being less common contributors [23].

## 4. Risk Factors and Associations with Other Cardiovascular and Systemic Conditions

All common cardiovascular risk factors, including smoking, age, hypertension and dyslipidaemia have been found to be associated with CMD [3], and are especially prevalent in patients with structural CMD. Not accidentally, these risk factors, in addition to diabetes, chronic kidney disease and obesity, are also associated with heart failure with preserved ejection fraction (HFpEF). In recent years, research has found links between HFpEF and CMD, although the cause-and-effect relationship between both entities has not yet been established [27]. The PROMIS-HFpEF study confirmed the presence of CMD in 75% of HFpEF patients and its association with markers of HF severity [28], with other studies highlighting >5-fold risk of HFpEF hospitalization and a higher rate of MACE and mortality in patients with co-existent CMD [29]. A cohort study by Rush et al. assessing the prevalence of endothelium-dependent and independent CMD in HFpEF has found a predominance of the latter type, which suggests that factors such as abnormal vascular remodelling, extrinsic vascular compression and microvascular rarefaction are more likely to be contributory than endothelial and vascular smooth muscle dysfunction [30], although endothelial dysfunction is associated with worse cardiovascular prognosis [31].

Importantly, the co-existence of CMD and obstructive coronary artery disease (CAD) in both chronic and acute scenarios must be recognised. Both CMD and CAD exert a synergistic effect on the myocardium, compounding myocardial ischaemia [27]. For example, epicardial CAD leads to reduced blood flow distal to stenosis, resulting in abnormal microvascular remodelling and vasodilator capacity, hindering collateral flow formation and contributing to stress-induced ischaemia [32]. Moreover, co-existent CMD may lead to an underestimation of the degree of severity of stenosis as measured by fractional flow reserve (FFR) [26]. The presence of CMD in patients with CAD may also explain why some patients do not derive symptomatic benefit from an apparently successful PCI. Importantly, raised index of microvascular resistance (IMR) values following revascularisation procedures despite an improvement in FFR were associated with adverse clinical outcomes [33]. Coronary microvascular obstruction (CMVO), an acute variant of CMD seen in patients with ST-elevation myocardial infarction, is still poorly understood but is associated with increased risk of left ventricular (LV) remodelling, heart failure and death [34,35,36]. Similarly, CMD was also found in >20% of cases of myocardial infarction with unobstructed coronary arteries (MINOCAs) [37]. It is unclear whether CMD has prognostic or causative implications or is a clinically insignificant bystander—in one stress perfusion cardiac magnetic resonance (CMR) study, abnormal stress perfusion was found in 63% of patients with MINOCA, but the areas corresponded with myocardial scar only in 75% of cases [38].

CMD presence has also been confirmed in studies of patients with hypertrophic cardiomyopathy (HCM), with evidence of impaired myocardial blood flow in both hypertrophied and non-hypertrophied myocardium [39] and reduced CFR in the subendocardium due to presumed extravascular compression [27]. Not only was severe CMD proven to exist in many asymptomatic HCM patients, but it has also been found to be important prognostically, with the degree of microvascular dysfunction being a strong and independent predictor of clinical deterioration and death [40]. Research utilising contemporary non-invasive imaging has also found an association between CMD and infiltrative cardiomyopathies, such as cardiac sarcoidosis, amyloidosis and Anderson–Fabry disease, with evidence of CMD being an early marker of disease in the latter two, which might present an optimal window for early treatment in these conditions [41,42,43,44].

In addition, inflammatory conditions, such as systemic lupus erythematosus or rheumatoid arthritis have also been found to be associated with microvascular angina, and the higher prevalence of these conditions in postmenopausal women than men might at least partly explain sex differences in CMD [3]. While the prevalence of CMD in women and men varies significantly between large cohort studies (34–66% in women and 14–60% in men) [45], it is uniformly associated with an increased risk of MACE in both sexes [46]. Interestingly, the commonest MACE seen in men is cardiovascular death, whereas heart failure-related hospital admission is most frequently seen in women, suggesting a different underlying pathophysiology [46]. A study assessing stress CMR-derived Myocardial Perfusion Reserve Index (MPRI)’s association with traditional cardiovascular risk factors has found that men with CMD tend to fit an atherosclerotic risk profile more so than women [45]. Indeed, a study comparing women suffering from angina with or without obstructive coronary disease has found that women with ANOCA are less likely to demonstrate conventional cardiovascular risk factors but more likely to report pre-menopausal migraines or rheumatic conditions [47]. The relationship between menopause and the prevalence of CMD among women has been frequently reported [48,49]. Studies have shown that oestrogen plays a protective role in coronary microvascular function by regulating endothelial and smooth muscle cells, indicating that changing oestrogen levels at the time of perimenopause may play an important role in CMD progression [50,51].

## 5. Diagnosis of Coronary Microvascular Dysfunction and Vasospastic Angina

### 5.1. Diagnostic Algorithm for Patients Presenting with ANOCA

**Figure 1:**
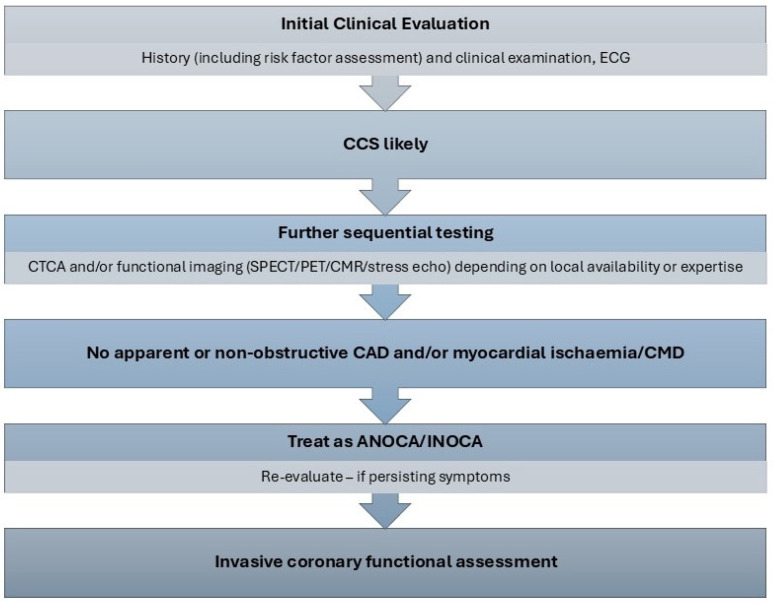
Diagnostic algorithm for ANOCA [3]. ANOCA—angina with no obstructive coronary artery disease, CAD—coronary artery disease, CCS—chronic coronary syndrome, CMD—coronary microvascular dysfunction, CMR—cardiac magnetic resonance imaging, CTCA—computed tomography coronary angiography, ECG—electrocardiogram, INOCA—ischaemia with no obstructive coronary artery disease, SPECT—single photon emission computed tomography, PET—positron emission tomography.

Both the European Association of Percutaneous Coronary Interventions (EAPCI) Expert Consensus Document [20] and the ESC guidelines [3] suggest a step-wise diagnostic approach to a patient presenting with anginal chest pain. The initial evaluation includes a clinical review of patient symptoms, physical examination and an electrocardiogram (ECG), with the aim of assessing risk factors for coronary disease and excluding non-cardiac disease or other alternative explanations for presentation. If clinical suspicion of angina is maintained, a non-invasive assessment should follow. This includes computed tomography coronary angiography (CTCA) or functional stress imaging. These tests can be performed in any sequence, depending on local availability and patient factors. In patients with no significant obstructive disease and/or no regional reversible ischaemia on functional testing, who have a significant burden of disease persistent despite medical treatment, invasive functional coronary assessment is recommended (ESC Class I recommendation) [3,20]. This process is summarised in Figure 1. Functional coronary assessment may allow identification of the underlying endotype of ANOCA, enabling stratified medical therapy as well as targeting modifiable risk factors, leading to improvement in symptoms and quality of life [14]. The clinical criteria for diagnosing both microvascular and vasospastic angina have been defined by the Coronary Vasomotor Disorders International Study (COVADIS) group and are presented in Table 1. These allow improvements in clinical diagnosis of affected patients and appropriate stratification of endotypes for subsequent research trials [52,53].

### 5.2. Invasive Methods for Diagnosis of CMD and VSA

Functional invasive coronary assessment provides accurate and reproducible evaluation of microvascular dysfunction with minimal additional resource use and at low risk of complications [54]. A complete study will involve the assessment of epicardial disease, as well as endothelium-dependent and independent CMD and VSA. Initial assessment of the coronary anatomy will involve a visual assessment of any epicardial disease and should include a physiological assessment of any equivocal epicardial stenoses (angiographic stenosis degree between 40 and 90%) with the use of FFR, or alternative resting indices (for example instantaneous wave-free ratio or resting full cycle ratio (iFR/RFR)) [55]. Patients with INOCA who have non-obstructive coronary atheroma are at increased risk of cardiovascular events, in addition to the risk conferred from microvascular myocardial ischaemia, and, thus, should receive intensive risk-reduction therapies, including lipid-lowering agents [56]. Absence of obstructive coronary atheroma with an FFR value < 0.8 in the presence of angina symptoms, especially but not necessarily in the context of ischaemia found on non-invasive testing, should prompt further investigation of coronary physiology. Microvascular coronary function testing can be performed utilising Doppler or thermodilution-based techniques.

#### 5.2.1. Doppler-Based Techniques

In the absence of obstructive coronary artery disease, reduced CFR is indicative of endothelium-independent coronary microvascular dysfunction. CFR is defined as a ratio of coronary blood flow (CBF) at hyperaemia versus rest. Historically, CBF was difficult to measure clinically, but Doppler-based techniques utilising a dual Doppler and pressure sensor coronary wire derive averaged peak flow velocity (APV) at rest and hyperaemia, which act as surrogate measurements of coronary blood flow [54]. Adenosine is selected as the most appropriate hyperaemic agent as it does not significantly influence the diameter of coronary arteries at peak hyperaemia [55] (albeit limiting this technique to the detection of endothelium-independent CMD only). Doppler-based CFR is, therefore, described as follows:

CFR = APV_hyperaemia_/APV_rest_.

Values above 2.5 are indicative of normal microvascular function, whereas values below 2.0 are defined as abnormal. Reduced CFR can however result from coronary atherosclerosis not immediately apparent as functionally significant, and, thus, microvascular resistance (MR) indices have been developed which may differentiate between diffuse coronary atheroma and microvascular dysfunction [57]. MR is defined as the ratio between myocardial perfusion pressure (which approximates distal coronary pressure (Pd)) and coronary blood flow. In the Doppler-based technique, the resulting index is called hyperaemic microvascular resistance (hMR) [18]:

hMR = Pd/APV.

Abnormal values are defined as hMR > 2.4 mmHg/(cm/s). A finding of isolated raised MR is still significant as those patients might benefit from CMD treatment, including statin therapy [14].

The doppler-based technique also allows the assessment of endothelial function by measurement of acetycholine flow reserve (AChFR). As described previously, the effect of ACh on coronary diameter depends on endothelial integrity and may result in either vasodilation or vasoconstriction—thus, volumetric coronary blood flow needs to be calculated, incorporating quantitative coronary angiography (QCA), and this equates to the following [54]:

_volumetric_CBF = 0.5 × π(APV)(vessel diameter/2)^2^.

ACh is infused either directly through a coronary guide catheter or through a dedicated infusion microcatheter, with the latter option being preferable as continuous aortic pressure measurements are available throughout the duration of ACh infusion. Various infusion protocols are in use—most commonly, either an infusion of three sequential concentrations, 0.182, 1.82 and 18.2 µg/mL at 1 mL/min for 2 min infusion periods, or an infusion of 18.2 µg/mL at 1 mL/min for 2 min followed by 2 mL/min for 2 min (when applied to LCA—for RCA the infusion dose or rate should be halved due to atrioventricular (AV) block concerns) [54]. Cine images are taken before and after each infusion and QCA is used to measure coronary diameter 5 mm distal to Doppler wire tip. AChFR is calculated as follows:

AchFR = _volumetric_CBF_ACh_/_volumetric_ CBF_rest_,

with values < 1.5 suggestive of coronary endothelial dysfunction and poorer long-term prognosis [58].

However, currently, Doppler sensor coronary wires are commercially unavailable and thus thermodilution techniques have emerged as the more commonly adopted.

#### 5.2.2. Thermodilution Techniques

A coronary microvascular study involving the thermodilution technique utilises a coronary wire with dual pressure and temperature sensors. Two approaches have been described—bolus and continuous thermodilution. Both techniques have been validated in the assessment of endothelium-independent coronary microvascular dysfunction [59,60].

Bolus thermodilution, a simpler and readily available method in most cardiac catheter laboratories, involves rapid sequential injections of 3 mL of room-temperature saline through a coronary guiding catheter. Mean transit time (Tmn) is calculated using dedicated software. An average of three values with less than 10% variation between them denotes resting mean transit time (Tmn_rest_). Hyperaemia is then induced using adenosine and hyperaemic mean transit time is derived (Tmn_hyperaemia_) in the same way. Coronary blood flow is defined as 1/Tmn, thus, from earlier equations, CFR is derived as:

CFR = Tmn_(rest)_/Tmn_(hyperaemia)_.

A value < 2.5 is considered abnormal and suggestive of endothelium-independent coronary microvascular dysfunction. The bolus thermodilution technique also allows measurement of microvascular resistance, termed IMR, defined as Pd/Tmn, with values > 25 being abnormal [54].

The bolus thermodilution technique has, however, been criticised as subject to substantial intrinsic variability (between 15 and 20%) [61], as well as inter- and intraoperator variability, and inferior correlation of CFR with the gold standard for myocardial perfusion quantification ([^15^O]H_2_O positron emission tomography (PET)) as compared to Doppler derived methods [62]. Bolus thermodilution methods also rely on infusion of adenosine, which may be contraindicated in some patients. The continuous thermodilution method allows direct measurement of absolute coronary flow (Q) at rest and hyperaemia and has been validated against PET imaging [59]. It provides a means of reducing operator dependence, with improved reproducibility of results as compared to bolus thermodilution [61,63]. This method involves the infusion of room-temperature saline at varying rates through a dedicated infusion microcatheter with four side ports. Infusion of saline at room temperature at a rate of 20 mL/min induces steady-state maximal hyperaemia equivalent to that obtained with adenosine [64], while infusion at 10 mL/min denotes absolute flow at rest [61]. CFR is thus derived as follows:

CFR = Q_hyperaemia_/Q_rest_,

and is a measure of endothelium-independent CMD.

Neither bolus nor continuous thermodilution methods allow assessment of endothelium-dependent CMD, although some centres are reporting the use of the continuous thermodilution method in the assessment of AChFR, expressed by the ratio of absolute flow under continuous infusion of varying concentrations of ACh vs. absolute flow at rest [65]. This method, however, requires further validation.

#### 5.2.3. Vasospasm Provocation

A complete invasive assessment of coronary vasomotor function also involves vasospasm assessment by intracoronary ACh bolus injections. This can be achieved by injecting incremental boluses of ACh up to 100 mcg into LCA over 20 s (or 50 mcg into RCA over 20 s), while closely monitoring the patient for signs and symptoms of ischaemia. Epicardial spasm is confirmed by observing a >90% reduction in coronary artery diameter on coronary angiography, associated with ischaemic ECG changes and symptoms [53]. Microvascular spasms can be diagnosed if typical symptoms and ECG change are seen without evidence of epicardial coronary artery spasm as per COVADIS criteria. However, continuous blood flow velocity assessment by coronary Doppler wire interrogation may allow for a more sensitive assessment of coronary microvascular spasm (with AChFR < 1.0, signifying reduced blood flow versus rest state), as alterations in coronary blood flow occur earlier in the ischaemic cascade than ECG change [54]. Coronary vasospasm should be reversed with the use of intracoronary nitrates to avoid prolonged ischaemia.

#### 5.2.4. Cardiac Catheter Laboratory Protocol for Invasive Coronary Function Assessment

**Figure 2:**
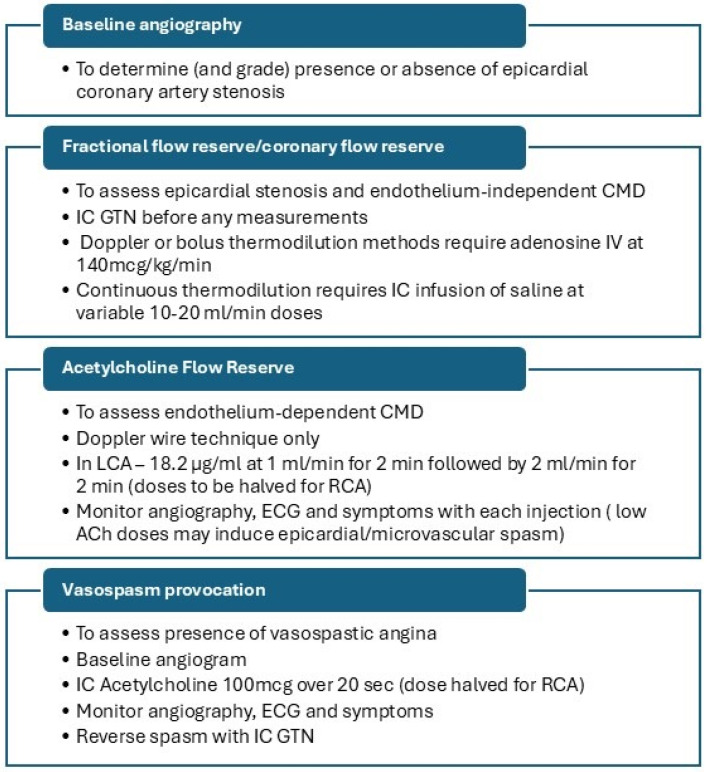
Invasive coronary function testing protocol. ACh—acetylcholine, CMD—coronary microvascular dysfunction, ECG—electrocardiogram, GTN—glyceryl trinitrate, IC—intracoronary, IV—intravenous, LCA—left coronary artery, RCA—right coronary artery.

The British Heart Foundation/National Institute for Health Research CMD (BHF/NIHR CMD) workgroup has standardised the cardiac catheter laboratory protocol (Figure 2), although variations of this protocol are also used in clinical practice.

**Figure 3:**
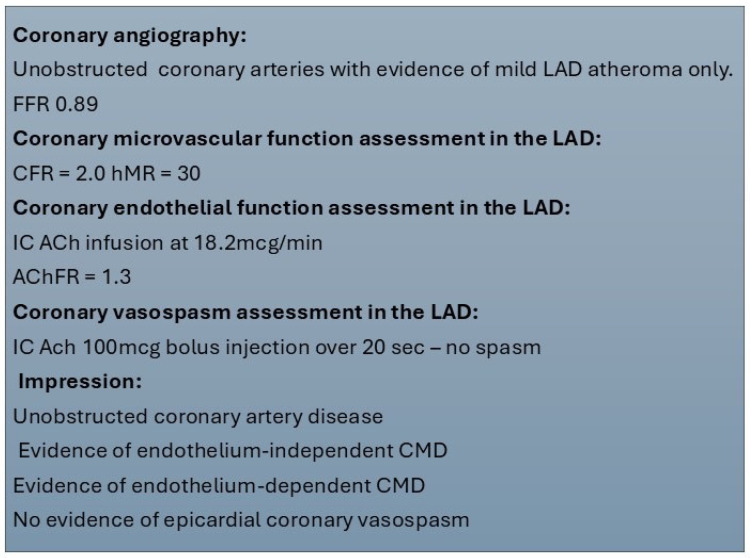
Sample report for invasive coronary function testing. ACh—acetylcholine, AChFR—acetylcholine flow reserve, CFR—coronary flow reserve, CMD—coronary microvascular dysfunction, FFR—fractional flow reserve, hMR—hyperaemic microvascular resistance, IC—intracoronary, LAD—left anterior descending.

Due to the unavailability of coronary Doppler wires, AChFR assessment is frequently omitted from this protocol, and some centres use both bolus and continuous thermodilution methods for endothelium-dependent CMD. Regardless of the protocol used, reporting of findings should be produced in a standardised manner. This should include a comment on epicardial coronary anatomy, coronary microvascular endothelium-dependent and independent function assessment, and vasospasm provocation results. A sample report is presented in Figure 3.

### 5.3. Non-Invasive Methods for CMD Diagnosis

In some patients, for example, in those for whom invasive testing may not be acceptable due to safety concerns or further rule-in tests following the discovery of non-obstructive coronary artery disease on CT coronary angiography is required, non-invasive imaging can be performed. Various methods have been described, including PET, CMR and Doppler echocardiography, each with their own advantages and disadvantages. These functional tests are currently awarded a class IIb recommendation in ESC guidelines for the investigation of persistently symptomatic patients with suspected ANOCA/INOCA [3].

Cardiac PET myocardial perfusion imaging at rest and hyperaemia, utilising endothelium-independent vasodilators, allows measurement of regional myocardial blood flow (MBF) in absolute terms. The quantification of MBF, and the derived myocardial flow reserve (MFR), extended the scope of myocardial perfusion imaging from assessment of ischaemia due to flow-limiting epicardial coronary disease to assessment of microvascular dysfunction, with evidence that impaired MBF and MPR as determined by PET in the absence of obstructive CAD is predictive of increased risk of MACE or death [66]. This method is considered the “gold standard” against which other non-invasive and invasive methods of coronary blood flow have been evaluated [27]. It has the benefit of assessing all myocardial territories simultaneously but is associated with significant radiation burden and is expensive and time-consuming.

Visual interpretation of myocardial perfusion defects on stress CMR is limited in the detection of CMD [67]; however, the development of the quantitative assessment of MBF or MPR by stress CMR now also provides diagnostic and prognostic value in patients with CMD [68,69,70]. Several studies have attempted validation of quantitative and semi-quantitative CMR against invasive methods of CMD assessment with various MBF and MPR cut-off values required for diagnosis [67,69,71]. CMR allows tissue characterisation as well as the assessment of all myocardial segments at once, and its high spatial resolution allows for the assessment of myocardial blood flow within specific layers of the myocardium, offering greater specificity for CMD [67]. The clinical use of quantitative stress CMR is, however, limited due to incomplete validation, the presence of imaging artefacts, a high cost and contraindications in specific patient populations [68].

Transthoracic Doppler echocardiography (TTDE) is another applicable method to assess coronary flow velocity reserve. This imaging technique, which utilises pulsed wave Doppler imaging of the proximal left anterior descending (LAD) segment at rest and with vasodilator hyperaemia, is widely available but hampered by technical difficulty and is limited to assessment of the LAD [68]. The data on the use of TTDE in non-obstructive CAD, although limited, show the reproducibility of this method in patients with CMD [72].

The invasive and non-invasive methods of CMD and VSA assessment are summarised in Table 2.

#### Novel Directions in Non-Invasive Assessment

One of the drawbacks of using non-invasive imaging techniques to diagnose CMD stems from the fact that the vasodilators used to achieve hyperaemia (adenosine or regadenoson) act only on the endothelium-independent pathways. Currently, endothelium-dependent CMD or vasospastic angina can only be diagnosed with intracoronary acetylcholine infusion; thus, complete coronary functional assessment necessitates invasive methods [20]. This is associated with a small risk of complications and increased initial cost. Thus, research focus is required to develop techniques which allow a complete, physiological assessment of all pathways involved in CMD and VSA pathophysiology.

Recently, there has been a resurgence of interest in exercise stress testing (EST). EST, historically validated against the presence of obstructive coronary artery disease, has been declassified in international guidelines due to apparent high false positive rates for obstructive CAD. However, as myocardial ischaemia can be present in the absence of obstructive CAD, recent studies have shown that, in patients with ANOCA, ischaemia seen on EST was highly specific for underlying ischaemic substrate, as determined by invasive functional coronary assessment [73,74]. While EST had poor sensitivity for the detection of both endothelium-dependent and independent CMD, it had excellent specificity for endothelium-dependent CMD, with low AChFR being the strongest predictor of ischaemia on EST [73]. The low sensitivity and high specificity of EST in detecting CMD as determined by invasive coronary blood flow measurements may be related to the fact that ECG changes occur further down the ischaemic cascade than changes in coronary blood flow. In the context of ANOCA, a negative EST does not rule out CMD, but a positive EST is highly suggestive of CMD (both endothelium and non-endothelium dependent) and thus could be used as a rule-in test in the diagnostic pathways.

Another novel technique focusing on physiological stress which is currently under investigation includes Oxygenation-Sensitive CMR (OS-CMR). In a healthy myocardium, stress decreases myocardial deoxyhaemoglobin concentration and increases OS-CMR signal intensity. Endothelium-dependent vasodilation can be induced using respiratory manoeuvres, such as hyperventilation followed by a breath-hold, and through the actions of carbon dioxide, a known physiological vasodilator. An inadequate response of coronary vasculature to vasodilatory stimuli (either adenosine or carbon dioxide) results in a higher concentration of deoxyhaemoglobin in the myocardium, detectable on OS-CMR [75]. In patients with ANOCA, OS-CMR has recently shown a heterogeneous coronary vascular response to a standardized vasoactive breathing manoeuvre with regionally reduced myocardial oxygenation, which may explain the presence of ischaemic symptoms [76]. This technique needs further validation but shows early promise in terms of the ability to non-invasively demonstrate and potentially differentiate between endothelium-dependent and independent CMD, allowing for precise non-invasive endotyping. The use of OS-CMR in non-invasive diagnosis of CMD is currently being investigated in a pilot CONCORD study (NCT06070662).

## 6. Management of Coronary Microvascular Dysfunction and Vasospastic Angina

Despite ongoing improvements in technology allowing invasive and non-invasive diagnostic options for CMD, robust data on therapeutic options is lacking, largely due to the paucity of large randomised controlled trials in patient groups clearly characterised by the ANOCA endotype. The majority of the trials of various therapeutic strategies have either taken place in the era before invasive testing for ANOCA was commonplace or simply included patients with evidence of chest pain with unobstructed coronaries, which contributes to heterogeneity of data and poor external validity of the results to current populations. A recent systematic review of all treatment studies in patients with ANOCA or CMD revealed that only a quarter of the studies enrolled patients who met contemporary COVADIS criteria for microvascular angina [77]. Sceptics of coronary vasoreactivity testing argue that subjecting patients to invasive diagnostics carries limited additional value over empirical antianginal therapy. However, as many as half of all patients with vasomotor angina proven on invasive testing are misdiagnosed as non-cardiac chest pain [78]. Arguably, the biggest contributor to poor patient outcomes and increased cardiovascular risk stems from inappropriate reassurance of patients with ANOCA and discontinuation of antianginal and prognostic therapies, such as statins or ACEi. Cross-over randomised controlled trial of ranolazine and amlodipine in patients with ANOCA (ChaMP-CMD) showed that only patients with impaired CFR derive benefit from anti-ischaemia therapy, which further supports the use of invasive diagnostic tools to avoid unnecessary treatments [79]. While no specific disease-modifying agents for CMD have been developed, evidence from CorMicA randomised controlled trial suggests that a stratified therapy based on specific ANOCA endotypes improves angina burden and quality of life [14], with invasive testing showing favourable cost-effectiveness [80]. A recent qualitative study addressed the psychological impact that the journey to diagnosis can have on patients with ANOCA [81]. International guidelines advocate a holistic approach to managing patients with suspected vasomotor angina, focusing on lifestyle factor modification, risk factor reduction and personalised antianginal therapy, as summarised in Table 3.

### 6.1. Lifestyle Factors Modification

Lifestyle advice is considered the cornerstone of management of CMD and VSA by international guidelines [3,12,20]. However, in practice, the delivery of lifestyle advice to patients with cardiovascular conditions both in primary and secondary care settings can be inconsistent, and the importance of lifestyle factors on disease management and progression is often overlooked by both the clinician and the patient [82,83,84,85]. Patients with CMD or VSA often have established coronary atherosclerosis and endothelial dysfunction where personalised counselling on lifestyle factors, including exercise, diet, weight management, smoking cessation and stress management strategies is mandated [3].

Exercise appears to enhance endothelium-dependent vasodilation of the microvasculature, improving myocardial perfusion, reducing anginal burden and improving functional capacity [86,87,88,89]. This effect appears most pronounced with high-intensity interval training (HIIT) [90]. Dietary interventions, particularly the Mediterranean diet, likewise have proven positive effects on the endothelium [91]. However, prior published work on diet and exercise interventions in CMD is very heterogeneous [88] and tested individual elements of lifestyle interventions separately. A recent pilot study of intense medical therapy and a supervised exercise regime lasting 12 weeks showed improvement in SAQ scores and MPR but was hampered by a significant drop-out rate (47%), highlighting challenges with cardiac rehabilitation adherence in this patient group [92]. A feasibility randomised controlled trial of a personalised rehabilitation programme combining diet and structured HIIT exercise in patients with CMD (MICROFIT) is currently underway and will assess the effects of such a programme on improvement in angina burden as well as functional capacity and myocardial perfusion (NCT06681896).

Psychological disorders may also play a significant role in vasomotor angina. Studies show a correlation between the prevalence of anxiety and depression in patients with ANOCA [93,94,95]. While a definite cause-and-effect relationship between mental state and angina is yet to be fully evaluated, the concept of Mental Stress-Induced Myocardial Ischaemia (MSIMI) has an established evidence base [96]. Patients with MSIMI appear to have impaired vasodilatory response to acetylcholine, implying endothelial dysfunction [97]. Importantly, in patients with stable coronary heart disease, MSIMI has been found to be associated with an increased risk of cardiovascular death or non-fatal myocardial infarction [98]. Psychotherapy or behavioural techniques targeting a patient’s response to mental stress and emotional regulation may, therefore, prove beneficial in angina management. There is some evidence that mindfulness-based stress reduction therapy can improve endothelial function in women with microvascular angina [99], although the evidence base in this area is generally sparse, with a Cochrane review of a highly heterogeneous sample of studies suggesting a modest to moderate effect of psychological interventions on patients with non-specific cardiac chest pain, which appeared to be limited to three months after the intervention [100].

Additionally, the impact of this chronic condition on patients’ quality of life, social interactions, employment and perception of control cannot be underestimated [81]. The maladaptive psychological response to chronic pain can potentiate pain perception and perpetuate potentially harmful behaviours, such as avoidance of exercise, obesity secondary to a sedentary lifestyle or smoking. In patients with chronic refractory angina, psychoeducational interventions focusing on challenging patients’ health beliefs and pragmatic goal setting have shown improvements in quality of life as well as a reduction in anxiety and depression scores and improved perceived control over anginal symptoms [101,102].

### 6.2. Risk Factor Reduction

Structural and functional changes in the coronary vasculature of patients with ANOCA are driven by the same cardiovascular risk factors as conventional coronary artery disease, such as hypertension, dyslipidaemia and diabetes. In patients with ANOCA, the presence of atherosclerosis is linked to an increased risk of MACE. In male patients with ANOCA, cardiovascular death is the most commonly occurring MACE [46], likely due to poorly managed risk factors. Therefore, it is essential to identify and manage these risk factors to improve patient outcomes.

The goal of antihypertensive therapy is to reduce microvascular remodelling. Blood pressure control can often be achieved through lifestyle changes (e.g., weight loss, exercise) and endotype-specific antianginal therapies (e.g., calcium channel blockers, beta-blockers). If needed, adding ACE inhibitors (ACEis) can be effective and well tolerated. ACEis may also benefit normotensive CMD patients by improving endothelial NO bioavailability and modulating microvascular tone [103,104]. A recent RCT in 61 women with CMD showed that quinapril improved both angina scores and coronary flow reserve, with the effect more pronounced in those with severe perfusion impairment [105]. This small trial forms the basis of the current ESC IIa recommendation to consider ACEis for symptom control in patients with endothelial dysfunction [3].

Importantly, statin therapy is known to reduce cardiovascular risk not only through direct reduction of LDL cholesterol but also through reduction of vascular inflammation and improvement of endothelial function [106]. It is known to be beneficial in patients with non-obstructive CAD [107], but there is also some evidence that it may confer a modest improvement in CFR in patients with CMD [108] and exercise tolerance, especially if used in combination with diltiazem [109].

Trials of both ACEi and statin use in patients with unobstructed coronary arteries have been small and heterogeneous, although both ACEi and statin therapy have been found to improve CFR in a recent meta-analysis [110]. Both statins and ACEis/ARBs have been shown to be associated with improved long-term clinical outcomes in observational trials of patients with VSA [111,112]. The hypothesis that a combination of ACEi/ARB and intensive statin therapy can reduce the risk of cardiovascular events, specifically in the cohort of female patients with ANOCA, will be tested in the randomized-controlled WARRIOR trial (NCT03417388) and provide much-needed high-quality evidence for use of these drugs in patients with ANOCA [113].

CMD is an early sign of diabetic cardiomyopathy, with microvascular dysfunction occurring before ventricular dysfunction. Functional CMD is more prevalent in non-insulin-dependent patients and the evidence seems to suggest that this disease can evolve into structural CMD as diabetes advances [114]. Poor glycaemic control is associated with CMD amongst women with diabetes and ANOCA [115]. Optimisation of diabetic control is, therefore, crucial in this cohort of patients. Interestingly, a small randomised controlled trial showed that metformin may improve endothelial function as well as exercise tolerance also in non-diabetic women with ANOCA [116], but the use of oral antiglycaemic agents, including sodium–glucose co-transporter 2 (SGLT2) inhibitors, in ANOCA populations needs to be further evaluated.

### 6.3. Pharmacological Therapies

Treatment of angina in ANOCA patients is challenging due to the lack of high-quality randomized trials addressing the heterogeneity of treated patients. Traditional antianginal medications are often ineffective, with many patients experiencing residual symptoms. Response to treatment varies by endotype—for example, beta-blockers are considered first-line for CMD, but may worsen vasospastic angina, while nitrates can be ineffective or exacerbate symptoms in CMD but are recommended as third-line therapy in VSA. These differences highlight the need for invasive endotyping in patients with refractory angina, where a personalised, stepwise approach is essential for optimal angina control.

#### 6.3.1. Beta-Blockers

Beta-blockers (BBs) decrease myocardial oxygen demand by reducing myocardial contractility and increase oxygen supply by increasing coronary perfusion during diastole. Early-generation BBs (e.g., propranolol) cause beta-mediated vasodilation whilst leaving unopposed alpha-mediated vasoconstriction, which may potentiate coronary vasospasm [117]. Third-generation BBs (e.g., carvedilol and nebivolol) exert an additional effect on CBF through NO-mediated vasodilation [118,119], an effect proven in hypertensive patients and thus potentially beneficial in patients with structural CMD [120,121]. Although some evidence seems to suggest that nebivolol can be beneficial in vasospastic angina [122], the international guidelines recommend vasodilatory BB use as a first-line therapy in CMD only [3,20]. This endorsement, however, is supported by surprisingly scanty and outdated evidence. A small cross-over randomised controlled trial (RCT) showed atenolol to significantly reduce chest pain episodes (over amlodipine and long-acting nitrates) in patients with cardiac syndrome X [123]. A small observational trial suggested that BB use was most associated with improved quality of life in patients with microvascular angina [124]. However, a most recent meta-analysis showed that BBs did not improve CFR in patients with unobstructed coronary arteries [110]. Further research is thus required to evaluate the role of BBs in symptom control as well as the prognosis of patients with ANOCA.

#### 6.3.2. Calcium Channel Blockers

Calcium channel blockers (CCBs) exert their antianginal effect through the reduction of oxygen consumption by negative inotropy, as well as a vasodilatory effect on VSMCs, which are implicated in the pathophysiology of coronary vasospasm [122]. There is conflicting evidence on the benefit of CCBs in CMD. An early case-control trial of 16 patients with microvascular angina did not show an improvement in CFR in patients who were administered intravenous (IV) diltiazem [125]. The ChaMP-CMD cross-over RCT did show an improvement in CFR with amlodipine in patients with CMD, but this was not reflected in an improvement in angina scores [79]. Interestingly, a recent meta-analysis showed CCBs to improve CFR in studies with a more prolonged (>6 months) follow-up period, suggesting that a longer duration of treatment may be required to exert effect [110]. The EDIT-CMD RCT, investigating a six-week-long course of diltiazem, did not find an improvement in CFR, anginal symptoms or quality of life of patients with ANOCA, but did show a reduction in the prevalence of epicardial coronary spasm through progression to microvascular spasm or no spasm [126]. CCBs have been extensively investigated at standard therapeutic doses and found to be effective in upwards of 80% of VSA patients [127]. Importantly, in observational studies, the use of CCBs in VSA has been associated with an improved prognosis [128,129], especially with benipidine [130]. The ESC guidelines give a Class I recommendation for the use of CCBs in VSA as the first line for symptom control and prevention of ischaemia and fatal complications and suggest the use of CCBs as the second line in CMD [3]. High doses of non-DHP CCBs may be required to control symptoms in severe VSA, with or without combination with DHP CCBs [20]; however, side effects may limit their use.

#### 6.3.3. Ranolazine

Ranolazine’s mode of action is incompletely understood, but it is believed to inhibit the late sodium current, reducing sodium and calcium overload in cardiomyocytes, which, in turn, improves myocardial relaxation and diastolic function. The largest RCT investigating a two-week course of ranolazine in 153 patients with CMD did not show an improvement in SAQ scores [131]. Ranolazine has since been investigated in a recent ChaMP-CMD RCT trial, showing improvement in both CFR and angina scores over 6 weeks [79], as well as the MARINA trial, which, in contrast, did not demonstrate an improvement in SAQ angina frequency score, microvascular function or functional capacity compared with placebo at 3 months [132]. The use of ranolazine has been evaluated in a 2024 meta-analysis and systematic review comprising RCTs, suggesting that it is associated with improvements in CFR, myocardial perfusion and angina scores in patients with CMD [133]. The study suggested that an extended duration of therapy may be needed for improvement in quality-of-life indices. The definite effect of ranolazine remains to be established through high-quality, large randomised controlled trials of sufficient therapy duration; however, it is nonetheless recommended as a third-line treatment for CMD in the international guidelines [3].

#### 6.3.4. Other (Trimetazidine, Ivabradine, Nicorandil, Nitrates)

Trimetazidine’s unusual action focuses on altering metabolism within cardiomyocytes by inhibiting fatty acid metabolism and stimulating myocardial glucose utilisation. This appears to have a cytoprotective effect on the myocardium during ischaemia [134]. The evidence for its use in CMD is limited—two placebo-controlled small RCTs showed an improvement in exercise time and degree of ischaemia on the exercise tolerance test following trimetazidine [135,136]. Nonetheless, as this drug does not exert any effect on a patient’s haemodynamic state, it is recommended as a further-line therapy for patients with CMD in the guidelines [3,20].

Ivabradine, a selective pacemaker channel blocker in the sinoatrial node, produces bradycardia without having an impact on myocardial contractility. This effect leads to increased coronary blood flow in diastole at rest and on exercise. The use of ivabradine in CMD remains poorly investigated, with one small RCT reporting improvement in SAQ angina scores and functional capacity [137], but no effect on myocardial perfusion in a meta-analysis [138].

Nicorandil exhibits a pleiotropic effect through dual properties of a nitrate and adenosine triphosphate (ATP)-sensitive potassium channel agonist, resulting in vasodilation due to NO synthesis as well as activation of the cyclic guanosine monophosphate (cGMP) signalling pathway in VSMCs [139]. Nicorandil may be beneficial in CMD due to cardiovascular protection from oxidative injury and systemic inflammation, as well as improved endothelial function [140]. A recent meta-analysis appears to suggest a positive effect of nicorandil on angina scores in patients with cardiac syndrome X, but a definite judgement could not be made due to publication bias and low quality of evidence [141]. Guidelines suggest the use of nicorandil as a further-line therapy in VSA and CMD [20].

Despite their effectiveness in patients with obstructive coronary artery disease, nitrates are poorly tolerated [142] and have a disappointing effect in CMD, which is thought to be secondary to a blood steal phenomenon where vasodilatation of the vessels within normally functioning myocardium reduces blood flow to dysfunctional territories [143]. This effect appears to extend to both long- and short-acting nitrate preparations. In VSA, however, short-acting nitrate plays an important role, although it appears to exhibit a greater effect in epicardial rather than microvascular spasms [144]. Observational data seem to suggest the prognostic disadvantage of nitrate therapy in addition to CCB [145], but it is unclear whether this represents a true effect or if long-acting nitrate use in VSA as an adjunct to CCBs is simply a marker of more severe disease. Long-acting nitrates are recommended as second-line therapy in epicardial coronary spasms, but tolerance as well as rebound vasoconstriction in therapy discontinuation may limit their use [146].

### 6.4. Non-Pharmacological Therapies

#### 6.4.1. Coronary Sinus Reducer

The coronary sinus reducer (CSR) device has emerged as a mechanical therapy for patients with refractory angina. This hourglass-shaped stent, implanted over a coronary sinus valve with 20% oversizing, exerts its anti-anginal effect after endothelialisation [147]. The exact mechanism of action of this device is not entirely known. It has been hypothesized to raise coronary venous pressure, which dilates subendocardial vessels, recruits capillaries, reduces microvascular resistance and redistributes blood from non-ischemic to ischaemic myocardium [148].

The CSR has primarily been studied in patients with left-sided epicardial coronary disease who are unsuitable for further revascularisation [149]. Clinical trials and multicentre registries demonstrate its efficacy in reducing angina severity and improving quality of life [150,151], earning it a Class IIb/Level of Evidence B indication in recent ESC guidelines [3].

The use of CSR for microvascular angina is less established, though case reports suggest it may reduce ischaemic burden, alleviate symptoms and improve microvascular function [152]. A recent blinded, sham-controlled crossover randomized trial of 20 patients with moderate/severe angina due to CMD showed that increased coronary venous pressure reduced microvascular resistance [153]. This was, however, a mechanistic study not designed to assess clinical outcomes. Additionally, in a group of eight patients with previous PCI and refractory angina with unobstructed coronaries, CSR implantation led to a significant improvement of symptoms and exercise tolerance, alongside evidence of ischemia reduction in a subgroup of these patients who underwent quantitative stress perfusion CMR [154]. These findings can only be interpreted as hypothesis-generating and will need further validation by adequately powered randomized trials.

#### 6.4.2. External Enhanced Counter Pulsation (EECP) Therapy

EECP is a non-invasive medical device the use of which has largely been tested in refractory angina secondary to obstructive coronary artery disease [155]. There is limited, although positive, evidence for its use in patients with CMD, including a single-centre observational trial which showed an improvement in exercise and functional capacity [156], and an RCT demonstrating an improvement in CFR and angina status in patients treated with EECP [157]. The use of these devices is, however, limited by patient discomfort, cost, accessibility and patient acceptability. Further research in this area is required, although a consideration to use this technique is recommended by the EAPCI consensus document in patients with refractory symptoms [20].

### 6.5. Other Therapies

Multiple other treatment strategies have been investigated previously, including tricyclic antidepressants [158], neuromodulation [159,160,161], rho-kinase inhibitors [162,163], hormone therapy [164,165], endothelin receptor antagonists [166], stem cell therapy [167,168] and phosphodiesterase inhibitors [169], some of which have shown promising effects in small trials. These treatment strategies are however not recommended in international guidelines as further research is required to provide evidence in terms of efficacy as well as the risk vs. benefit ratio of these therapies in patients with ANOCA.

## 7. Conclusions

Coronary microcirculation dysfunction, as fascinating an entity as it may be, is a major global health problem which impacts both patients and health systems. Current technology allows us to better study the disease, both via invasive and non-invasive modalities. As our knowledge expands and we better understand the pathophysiologic pathways of the disease, we will be able to provide patients with a more personalized treatment. The current pharmacological therapies have a limited evidence base but with improvements in diagnostic rates and endotype characterisation; well-designed and targeted randomised controlled trials can now be undertaken to help guide treatment. Novel interventional therapies are still under investigation and might be an addition to established pharmacotherapies in the cardiologist’s armamentarium.

## Figures and Tables

**Table 1:** Standardised diagnostic criteria for microvascular angina and vasospastic angina, derived by COVADIS working group [52,53].

Standardised Diagnostic Criteria for Microvascular and Vasospastic Angina	
Microvascular Angina ^a^	Vasospastic Angina ^b^
**1. Symptoms of myocardial ischaemia** Effort and/or rest angina or angina equivalents (i.e., breathlessness)	**1. Nitrate-responsive angina in addition to at least one of the following:** (a) Rest angina—especially between night and early morning (b) Marked diurnal variation in exercise tolerance (c) Angina precipitated by hyperventilation (d) Response to calcium channel blockers (but not beta-blockers)
**2. Absence of obstructive epicardial CAD** (<50% stenosis or FFR < 0.80) assessed on either CT coronary angiogram or invasive coronary angiography	**2. Transient ischaemic ECG changes during a spontaneous episode** in at least two contiguous leads (ST segment elevation or depression ≥ 0.1 mV, new negative U waves)
**3. Objective evidence of myocardial ischaemia** e.g., ischemic ECG changes during an episode of chest pain, stress-induced chest pain and/or ischemic ECG changes in the presence or absence of transient/reversible abnormal myocardial perfusion and/or wall motion abnormality	**3. Coronary artery spasm**—defined as >90% coronary lumen constriction with angina and ischaemic ECG changes (spontaneous or in response to a provocative agent, e.g., acetylcholine, ergonovine or hyperventilation).
**4. Evidence of impaired coronary microvascular function** e.g., impaired coronary flow reserve (cut-off values between ≤2.0 and ≤2.5), microvascular spasm (reproduction of symptoms, ischemic ECG shifts but no epicardial spasm during Ach testing), abnormal coronary microvascular resistance indices (e.g., IMR > 25), coronary slow flow (TIMI frame count > 25)	

^a^ Diagnosis of microvascular angina is only confirmed if all four criteria are met. Diagnosis of microvascular angina is suspected if patient fulfils criteria 1 and 2 but only criterion 3 or 4 alone. ^b^ Diagnosis of vasospastic angina is confirmed if condition 1 and either 2 or 3 are fulfilled. Diagnosis of vasospastic angina is suspected if condition 1 is met but conditions 2 or 3 are equivocal. CAD—coronary artery disease, CT—computed tomography, ECG—electrocardiogram, IMR—index of microvascular resistance, FFR—fractional flow reserve, TIMI—thrombolysis in myocardial infarction.

**Table 2:** Invasive and non-invasive methods of CMD and VSA diagnosis.

Method	Technique	Hyperaemic/Vasoactive Agent	Advantages	Disadvantages
Invasive methods				
Intracoronary Doppler flow-pressure wire	Measurement of averaged coronary peak flow velocity	Adenosine Acetylcholine	Gold standard invasive method of CMD assessment Ability to assess endothelium-independent and endothelium-dependent function Ability to assess FFR to rule out obstructive CAD	Currently commercially unavailable Requires adenosine infusion which may be contraindicated in severe lung disease Endothelium-dependent CMD assessment relies on QCA measurements of coronary artery diameter, potentially introducing error
Bolus thermodilution method	Estimation of coronary blood flow through measurement of mean transit time in response to boluses of saline at rest and hyperaemia	Adenosine	Simple and quick Ability to measure FFR to rule out obstructive CAD	Significant intrinsic variability and inter- and intraoperator variability Worse correlation with PET (non-invasive gold standard) than Doppler method Endothelium-independent CMD assessment only
Continuous thermodilution method	Quantification of absolute flow through infusion of saline at variable rates through a dedicated microcatheter	Saline	Reproducible assessment of endothelium-independent CMD as there is minimal operator interference No need for adenosine infusion to induce hyperaemia Method for assessing endothelium dependent CMD is in development Ability to assess obstructive CAD with FFR	Requires use of a dedicated infusion microcatheter Longer procedure than bolus thermodilution method
Vasospasm provocation testing	Assessment of coronary artery diameter, symptoms and ECG changes in response to intracoronary acetylcholine or ergonovine infusion	Acetylcholine Ergonovine	The only method available to assess epicardial coronary spasm Easy to assess No additional equipment required	Risk of complications (arrhythmias/persistent spasm leading to myocardial infarction or death) Drug availability
**Non-invasive methods**				
Cardiac PET	Dynamic rest and stress myocardial perfusion imaging	Adenosine, regadenoson, dipyridamole	Gold standard for non-invasive CMD assessment Ability to quantitatively assess global myocardial perfusion	Ability to assess endothelium-independent CMD only Radiation burden Limited spatial resolution Limited availability and high cost Contraindicated in specific patient populations, e.g., severe asthma or heart block
Cardiac MRI	Quantitative or semiquantitative rest and stress assessment of myocardial perfusion	Adenosine, regadenoson	No radiation exposure Ability to assess global myocardial perfusion Spatial resolution allows assessment of specific layers of myocardium increasing specificity for CMD	Incomplete validation of quantitative stress perfusion MRI Contraindicated in specific patient populations (metallic implants, renal dysfunction, asthma, heart block) Ability to assess endothelium independent CMD only Presence of artefacts Limited availability and high cost
Echocardiography	Transthoracic Doppler echocardiography assessment of flow in left anterior descending artery at rest and hyperaemia	Adenosine, dipyridamole, regadenoson	Widely available and inexpensive method No radiation exposure	Highly operator and patient factor dependent Ability to assess endothelium-independent CMD only Hyperaemic agents contraindicated in specific patient populations (e.g., severe asthma/heart block) Assessment limited to the LAD territory.

CAD—coronary artery disease, CMD—coronary microvascular dysfunction, FFR—fractional flow reserve, MRI—magnetic resonance imaging, LAD—left anterior descending artery, PET—positron emission tomography, QCA—quantitative coronary angiography.

**Table 3:** International guideline-directed strategies for holistic management of ANOCA/INOCA.

**General management principles ^ab^**		
Improved nutrition Exercise rehabilitation Weight management Smoking cessation Mental wellbeing management		
**Risk factor modification ^ab^**		
Hyperlipidaemia management Blood pressure control Glycaemic control		
**Endotype specific antianginal management**		
**Endotype**	**Treatment**	**Class/level of evidence**
Coronary Microvascular Dysfunction	Betablockers (nebivolol)	Class IIa, Level of Evidence B ^ab^
	Calcium channel blockers (DHP and non-DHP)	Class IIa, Level of Evidence B ^ab^
	Ranolazine	Class IIa, Level of Evidence B ^ab^
	Trimetazidine	Class IIa, Level of Evidence B ^ab^
Vasospastic angina	Calcium channel blocker	Class I, Level of Evidence A ^ab^
	2nd Calcium channel blocker	
	Long-acting nitrate	Class IIa, Level of Evidence B ^ab^
	Nicorandil	Expert opinion ^b^
Endothelial dysfunction	Angiotensin-converting enzyme inhibitors	Class IIa, Level of Evidence B ^a^
Mixed endotypes	Nitrates Calcium Channel blockers Other vasodilators	Class IIb, Level of Evidence B ^ab^
**Advanced non-pharmacological therapies**		
Consider Enhanced Extracorporeal Counterpulsation therapy ^b^		

^a^ Derived from the 2024 Chronic Coronary Syndrome ESC guidelines [3]. ^b^ Derived from the 2021 EAPCI Consensus document on ischaemia with non-obstructive coronary artery disease [20]. DHP—dihydropyridine.

## Abbreviations

The following abbreviations are used in this manuscript:

ACh	Acetylcholine
AChFR	Acetylcholine Flow Reserve
ACEi	Angiotensin Converting Enzyme inhibitor
ANOCA	Angina with Non-Obstructed Coronary Arteries
APV	Averaged Pulsed Velocities
ARB	Angiotensin II Receptor Blocker
ATP	Adenosine Triphosphate
AV	Atrio-ventricular
BB	Beta-Blocker
BHF	British Heart Foundation
CAD	Coronary Artery Disease
CBF	Coronary Blood Flow
CCB	Calcium Channel Blocker
CCS	Chronic Coronary Syndromes
CFR	Coronary Flow Reserve
cGMP	Cyclic Guanosine Monophosphate
CMD	Coronary Microvascular Dysfunction
CMVO	Coronary Microvascular Obstruction
COVADIS	Coronary Vascular Disorders International Study Group
CSR	Coronary Sinus Reducer
CT	Computed Tomography
DHP	Dihydropyridine
EAPCI	European Association of Percutaneous Coronary Intervention
ECG	Electrocardiogram
EECP	Enhanced External Counterpulsation
ESC	European Society of Cardiology
EST	Exercise Stress Test
FFR	Fractional Flow Reserve
GTN	Glyceryl Trinatrate
HCM	Hypertrophic Cardiomyopathy
HFpEF	Heart Failure with Preserved Ejection Fraction
HIIT	High Intensity Interval Training
hMR	Hyperaemic Microvascular Resistance
IC	Intra-coronary
iFR	Instantaneous wave-free ratio
IMR	Index of Microvascular Resistance
INOCA	Ischaemia with Non-Obstructed Coronary Arteries
IV	Intravenous
MACE	Major Adverse Cardiovascular Events
MBF	Myocardial Blood Flow
MINOCA	Myocardial Infarction with Non-Obstructed Coronary Arteries
MMR	Minimal Microvascular Resistance
MPR	Myocardial Perfusion Reserve
MR	Microvascular Resistance
MRI/CMR	Magnetic Resonance Imaging/Cardiac Magnetic Resonance Imaging
MSIMI	Mental State Induced Myocardial Ischaemia
NIHR	National Institute of Health Research
LAD	Left Anterior Descending
LCA	Left Coronary Artery
LDL	Low Density Lipoprotein
LV	Left Ventricle
NO	Nitric Oxide
PCI	Percutaneous Coronary Intervention
PET	Positron Emission Tomography
OS-CMR	Oxygenation-Sensitive Cardiac Magnetic Resonance Imaging
Q	Absolute Coronary Flow
QCA	Quantitative Coronary Angiography
RCA	Right Coronary Artery
RCT	Randomised Controlled Trial
RFR	Resting full cycle Ratio
SAQ	Seattle Angina Questionnaire
SGLT2	Sodium-Glucose Cotransporter 2
SPECT	Single Photon Emission Computed Tomography
TIMI	Thrombolysis in Myocardial Infarction
Tmn	Mean transit time
TTDE	Transthoracic Tissue Doppler Echocardiography
VSA	Vasospastic Angina
VSMC	Vascular Smooth Muscle Cells
